# Dietary Omega-3 Fatty Acid Dampens Allergic Rhinitis via Eosinophilic Production of the Anti-Allergic Lipid Mediator 15-Hydroxyeicosapentaenoic Acid in Mice

**DOI:** 10.3390/nu11122868

**Published:** 2019-11-22

**Authors:** Kento Sawane, Takahiro Nagatake, Koji Hosomi, So-ichiro Hirata, Jun Adachi, Yuichi Abe, Junko Isoyama, Hidehiko Suzuki, Ayu Matsunaga, Satoshi Fukumitsu, Kazuhiko Aida, Takeshi Tomonaga, Makoto Arita, Jun Kunisawa

**Affiliations:** 1 Nippon Flour Mills Co., Ltd., Innovation Center, Kanagawa 243-0041, Japan; 2 Laboratory of Vaccine Materials, Center for Vaccine and Adjuvant Research and Laboratory of Gut Environmental System, National Institutes of Biomedical Innovation, Health and Nutrition (NIBIOHN), Osaka 567-0085, Japan; 3 Graduate School of Pharmaceutical Sciences, Osaka University, Osaka 565-0871, Japan; 4 Department of Microbiology and Immunology, Kobe University Graduate School of Medicine, Hyogo 650-0017, Japan; 5 Laboratory of Proteome Research, NIBIOHN, Osaka 567-0085, Japan; 6 Division of Molecular Diagnosis, Aichi Cancer Center Research Institute, Nagoya 464-8681, Japan; 7 Collaborative Graduate School Program, University of Tsukuba, Ibaraki 305-8577, Japan; 8 Division of Physiological Chemistry and Metabolism, Graduate School of Pharmaceutical Sciences, Keio University, Tokyo 105-8512, Japan; 9 Laboratory for Metabolomics, RIKEN Center for Integrative Medical Sciences, Kanagawa 230-0045, Japan; 10 Cellular and Molecular Epigenetics Laboratory, Graduate School of Medical Life Science, Yokohama City University, Kanagawa 230-0045, Japan; 11 Graduate School of Medicine and Graduate School of Dentistry, Osaka University, Osaka 565-0871, Japan; 12 Division of Mucosal Immunology, Department of Microbiology and Immunology and International Research and Development Center for Mucosal Vaccines, the Institute of Medical Science, the University of Tokyo, Tokyo 108-8639, Japan

**Keywords:** omega-3 fatty acids, nasal allergy, eosinophil, lipid metabolism, PPAR

## Abstract

The metabolism and generation of bioactive lipid mediators are key events in the exertion of the beneficial effects of dietary omega-3 fatty acids in the regulation of allergic inflammation. Here, we found that dietary linseed oil, which contains high amounts of alpha-linolenic acid (ALA) dampened allergic rhinitis through eosinophilic production of 15-hydroxyeicosapentaenoic acid (15-HEPE), a metabolite of eicosapentaenoic acid (EPA). Lipidomic analysis revealed that 15-HEPE was particularly accumulated in the nasal passage of linseed oil-fed mice after the development of allergic rhinitis with the increasing number of eosinophils. Indeed, the conversion of EPA to 15-HEPE was mediated by the 15-lipoxygenase activity of eosinophils. Intranasal injection of 15-HEPE dampened allergic symptoms by inhibiting mast cell degranulation, which was mediated by the action of peroxisome proliferator-activated receptor gamma. These findings identify 15-HEPE as a novel EPA-derived, and eosinophil-dependent anti-allergic metabolite, and provide a preventive and therapeutic strategy against allergic rhinitis.

## 1. Introduction

Allergic rhinitis is among the most common allergic diseases and is increasing in prevalence globally [1,2]. Patients with perennial allergic rhinitis continuously suffer from symptoms such as sneezing, itchiness, and rhinorrhea; they, therefore, have to avoid contact with allergens, causing a heavy burden and lifestyle limitations [2].

The initiation of allergic rhinitis is dependent on the function of antigen-specific immunoglobulin (Ig) E [3,4]. When exposed to an antigen for the first time, antigen-presenting cells take up the antigen and induce the differentiation and proliferation of T cells to type 2 helper T (Th2) cells. Th2 cells induce Ig class-switching to IgE on B cells and stimulate differentiation to antigen-specific IgE-producing plasma cells through the secretion of Th2 cytokines such as interleukin (IL)-4, IL-5, and IL-13 [3]. Antigen-specific IgE secreted by plasma cells is bound to the cell surface of mast cells via Fc epsilon receptor 1 (FcεRI), and when the individual is re-exposed to the antigen, it initiates FcεRI cross-linking and induces mast cell degranulation [3]. Degranulated mast cells secrete chemical mediators, including histamine and lipid mediators, that induce the nasal responses of allergic rhinitis, including sneezing, itchiness, and rhinorrhea [5]. Given these mechanistic processes, promising strategies to control allergic symptoms include the inhibition of Th2 differentiation, IgE production, mast cell degranulation, and the interaction between chemical mediators and their receptors.

Previous studies about the association between the development of allergic diseases and dietary nutrition indicate the involvement of dietary fatty acid (FA) in the acceleration and/or inhibition of allergic responses [6,7]. Dietary oils contain various FAs, but their composition is dependent on the materials from which the oils are extracted as well as the extraction process. FA is characterized by the presence or absence of a carbon double bond in its structure; saturated FAs (palmitic acid, stearic acid, etc.) lack a carbon double bond, and unsaturated FAs contain at least one carbon double bond. Among unsaturated FAs, omega-3 FAs (e.g., alpha-linolenic acid [ALA], eicosapentaenoic acid [EPA], and docosahexaenoic acid [DHA]) and omega-6 FAs (e.g., linoleic acid [LA] and arachidonic acid [ARA]) are categorized as essential FAs. Various types of bioactivity of dietary essential FAs have been reported in studies of health and diseases, including immunity, allergy, and inflammation [6,8]. Human studies, for example, have shown an association between the quality of dietary FAs and the incidence of allergic diseases [9]. Animal studies likewise indicate that the quality of dietary FAs is a critical determinant of the development and severity of allergic symptoms [10,11]. We previously showed that dietary linseed oil (also called flaxseed oil), which contains high amounts of ALA, ameliorates egg-derived ovalbumin (OVA)-induced food allergy in mice [12]. Another report suggested that pollen-derived antigen-induced allergic conjunctivitis can be alleviated by dietary linseed oil [13]. These studies demonstrate the importance of dietary FA quality in the regulation of allergic responses and the contribution of omega-3 FA-rich linseed oil to the inhibition of allergic symptoms.

Dietary FAs are not only an energy source [14] but also the substrate of bioactive lipid metabolites [15]. FAs usually exist as components of triglycerides and cell membrane phospholipids in vivo. Under certain circumstances, including cell activation, FAs are degraded from membrane phospholipids by phospholipases. Free FAs can be further converted to various metabolites by oxygenate enzymes (e.g., lipoxygenase [LOX], cyclooxygenase [COX], and cytochrome P450 [CYP]), and the resulting compounds can exacerbate and/or ameliorate allergic diseases [15]. Recently, the application of a new method of liquid chromatography coupled with tandem mass spectrometry (LC–MS/MS) revealed that tremendous numbers of metabolites are generated from dietary FAs, including omega-6 and omega-3 essential FAs [16]. Further biological studies found novel functions of these FA metabolites in immunity, allergy, and inflammation [16]. In particular, recent studies have uncovered that omega-3 FA-derived metabolites have anti-allergic functions [9]. For example, EPA-derived resolvin E1 promotes the resolution of allergic lung inflammation [17], and DHA-derived resolvin D1 reduces histamine responses in the eye and regulates conjunctival goblet cell secretion [18]. These studies indicate that omega-3 FA-derived metabolites have potential to regulate inflammation and allergic disease. The accumulation of omega-3 FA-derived metabolites is increased upon the intake of dietary linseed oil. In our previous report, 17,18-epoxyeicosatetraenoic acid (17,18-EpETE), which is converted from EPA by CYPs, was among the main metabolites that accumulated in the mouse large intestine, and it indeed exerted anti-allergic effects in a food allergy model [12]. However, it remains unclear whether dietary linseed oil has anti-allergic effects at sites other than the intestine, such as the respiratory mucosal compartment, and whether unique anti-allergic FA metabolites accumulate in nasal tissue.

In this study, we evaluated the effect of dietary linseed oil on allergic rhinitis in the upper respiratory tract by using an OVA-induced nasal allergy model in mice.

## 2. Materials and Methods

### 2.1. Mice and Experimental Diet

Female C57BL/6J mice (age, 6–7 weeks) were purchased from Japan SLC. Animals were maintained in the specific pathogen-free facility of the National Institutes of Biomedical Innovation, Health, and Nutrition (NIBIOHN). Mice were maintained for 2 months on diets composed of chemically defined materials and supplemented with 4% of each dietary oil (Table S1) (Oriental Yeast, Tokyo, Japan) before the induction of allergic rhinitis. The fatty acid composition of dietary oils is shown in Figure S1. All animal experiments were conducted in accordance with the guidelines of the Animal Care and Use Committee and the Committee on the Ethics of Animal Experiments at NIBIOHN (DS27-47 and DSR01-2).

### 2.2. Induction of Allergic Rhinitis

Allergic rhinitis was induced as described previously, with modifications [19,20,21,22]. In brief, mice were sensitized by intraperitoneal injection of 5 μg of OVA (Sigma-Aldrich, St. Louis, MO, USA), with 1 mg of alum hydroxide (Thermo Fisher Scientific, Waltham, MA, USA) in 200 μL of phosphate-buffered saline (PBS) (Nacalai Tesque, Kyoto, Japan). After 7 days, awake mice were intranasally challenged with either 250 μg of OVA in 20 μL of PBS or PBS alone as a vehicle control for 4 consecutive days. Allergic symptoms were assessed by counting the number of sneezing behaviors in a 5 min period after the nasal challenge. Soon after nasal administration, mice showed sneezing, which could be recognized by sound and neck movement. The enumeration of sneezing numbers was performed in a quiet room in order to reduce noises and to detect sneezing sound clearly. The measurement was conducted one by one and the mouse was put in a cage for a 5 min period after the nasal challenge.

### 2.3. Administration of Reagents to Mice

The 15-hydroxyeicosapentaenoic acid (15-HEPE) was purchased from Cayman Chemical (Ann Arbor, MI, USA). Mice were intranasally treated with either 10 ng of 15-HEPE in 20 μL of PBS or 0.5% (*vol*/*vol*) ethanol in PBS as a vehicle control, 5 min before the nasal challenge. To evaluate the ability of 15-HEPE to prevent the development of allergic rhinitis, lipid administration began on the first day of the nasal challenge. To evaluate the therapeutic effect of 15-HEPE, lipid administration was started from the fifth day of the nasal challenge.

GW9662 (Abcam, Cambridge, United Kingdom), a peroxisome proliferator-activated receptor gamma (PPARγ) antagonist, was dissolved in PBS containing 0.5% (*vol*/*vol*) ethanol. PBS with 0.5% ethanol was used as a mock-treatment control. GW9662 solution or the mock-treatment was intraperitoneally injected daily at 1 mg/kg of body weight in a 200 μL volume, at 30 min before nasal administration of the lipid solution.

PD146176 (Cayman Chemical) was dissolved in PBS containing 1% (*vol*/*vol*) dimethyl sulfoxide (DMSO). PBS with 1% (*vol*/*vol*) DMSO was used as a mock-treatment control. This solution was intraperitoneally injected daily at 1 mg/kg of body weight in a 200 μL volume, at 30 min before the nasal challenge.

The mixture of anti-C-C motif chemokine ligand (CCL) 11 antibody (24 μg: Biolegend, San Diego, CA, USA) and anti-IL-5 antibody (3 μg: Biolegend) in a 100 μL volume was intravenously administrated to the allergic rhinitis mouse model on the first and third days of the nasal challenge. Rat immunoglobulin G_1_ (IgG_1_) (27 μg: Biolegend) was used as an isotype control [23].

### 2.4. Tissue Collection and Preparation of a Single Cell Suspension

Nasal passage (NP) cells were collected from mice as previously reported, with modifications [19,20]. The cell suspension was filtered through 100 μm cell strainers (BD Biosciences, Franklin Lakes, NJ, USA).

### 2.5. Flow Cytometry

Flow cytometric analysis was performed as described previously, with modifications [24]. In brief, the cell suspension in 2% newborn calf serum (NCS) PBS was stained with an anti-CD16/32 antibody (TruStain fcX) (Biolegend) to avoid non-specific staining. After being washed with 2% NCS PBS, the cells were further stained with the following antibodies: FITC-anti-Ly6G (Biolegend), FITC-anti-CD63 (gift from Dr. Kurashima, The University of Tokyo) [25], APC-Cy7-anti-CD11b (Biolegend), APC-anti-FcεRI (eBioscience), PE-anti-c-kit (BD Biosciences), PE-Cy7-anti-CD45 (Biolegend), BV421-anti-CD45 (Biolegend), and BV421-anti-Siglec-F (Biolegend). Dead cells were detected by using 7-AAD (Biolegend) and were excluded from the analysis. Flow cytometry analysis was performed by using MACSQuant (Miltenyi Biotec, Bergisch Gladbach, Germany) or FACSAria (BD Biosciences). Data were analyzed by using FlowJo 9.9 (Tree Star, Ashland, OR, USA).

### 2.6. OVA-Specific IgE ELISA

Plasma was collected 5 min after the last nasal challenge and heparinized. OVA-specific IgE levels in the plasma were determined by using an ELISA kit (DS Pharma, Osaka, Japan) according to the manufacturer’s protocol.

### 2.7. Mast Cell Degranulation Assay

Mouse bone marrow-derived mast cells (BMMCs) were prepared and degranulation was induced as previously reported [25] In brief, bone marrow cells were prepared from the femur and tibia of 8-week-old C57BL/6J wild-type mice and were cultured in RPMI1640 (Sigma-Aldrich) supplemented with 10% FBS, 100 IU/mL penicillin plus 100 μg/mL streptomycin (Nacalai Tesque), 1 mM 4-(2-hydroxyethyl)-1-piperazineethanesulfonic acid (HEPES) (Nacalai Tesque), 1 mM sodium pyruvate (Nacalai Tesque), and 5 ng/mL mouse recombinant IL-3 (PeproTech, Rocky Hill, CT, USA) for 4 weeks. After that, 50 ng/mL stem cell factor (SCF) (PeproTech) was added to the culture medium, and the cells were incubated for 2 to 4 weeks. The efficacy and percentage of differentiation to BMMCs were confirmed by flow cytometry as the FcεRI+, c-Kit+, CD45+ population; a > 90% pure population was used for the degranulation assay.

For the degranulation assay, BMMCs were sensitized with 0.2 mg/mL anti-dinitrophenyl (DNP)-IgE (Sigma-Aldrich) for 24 h. Cells were then washed and stimulated with 100 ng/mL DNP-BSA (LSL, Tokyo, Japan) at 37 °C for 30 min. To assess the effect of lipid mediators on degranulation, 15-HEPE (100 nM) or 0.5% (*vol*/*vol*) EtOH in PBS as a vehicle control was added 30 min before stimulation with DNP-BSA. Degranulation of BMMCs was evaluated by using flow cytometry and staining the BMMCs with CD63 as a marker for degranulation.

### 2.8. Culture of Bone Marrow-Derived Neutrophils and Eosinophils

Neutrophils were collected from bone marrow cells of female C57BL/6J mice as previously reported [26]. Bone marrow cells were suspended in RPMI 1640 medium containing 2% NCS and carefully added onto 62% Percoll (GE Healthcare, Chicago, IL, USA) in RPMI 1640 medium. After centrifugation at 1000× *g* for 20 min at 25 °C, neutrophils were precipitated.

Eosinophils were prepared by differentiation from mouse bone marrow cells as previously reported [27]. Briefly, mouse bone marrow cells were cultured at 1.0 × 106 cells/mL in medium containing RPMI 1640 with 20% FBS, 100 IU/mL penicillin plus 10 μg/mL streptomycin (Nacalai Tesque), 25 mM HEPES (Nacalai Tesque), 1× nonessential amino acids (Nacalai Tesque), 1 mM sodium pyruvate (Nacalai Tesque) and 50 μM 2-ME (Thermo Fisher Scientific). From day 0 through 4, 100 ng/mL stem cell factor (SCF; [PeproTech]) and 100 ng/mL FLT3 ligand (PeproTech) were supplemented. On day 4, the medium with SCF and FLT3 ligand was replaced to the medium containing 10 ng/mL mouse recombinant IL-5 (Peprotech). From day 8 through 10, the cells were transferred to a new flask and the medium was changed. The cell concentration was adjusted to 1.0 × 106 cells/mL every day. On day 12, eosinophil differentiation was assessed by FACS staining with BV421-anti-Siglec-F and the cells were used for experiments between day 12 and day 19.

### 2.9. Lipid Metabolism of Cultured Neutrophils and Eosinophils

A lipid metabolism assay was performed as previously reported with modification [28]. Neutrophils or eosinophils were suspended in RPMI 1640 at 1.0 × 10^6^ cells/mL. A lipid production assay was performed with the addition of 1 μM EPA or ARA, together with 2 μM calcium ionophore in the culture medium. After 30 min, the reaction was stopped by adding 2 times the amount of ice-cold methanol to the medium.

### 2.10. Lipid Extraction from Cells, Culture Supernatant, and Plasma

Lipid extraction was performed as previously reported [29]. Cells were suspended in PBS and transferred to a polypropylene tube. After centrifugation to remove the PBS, methanol was added to extract the lipids. For culture supernatant and plasma, 9 volumes of methanol were used for the extraction. After centrifugation at 1600× *g* for 10 min at 4 °C, the supernatant was collected and diluted to 50% methanol. Solid-phase extraction was then performed by using a Mono Spin C18-AX cartridge (GL Science, Tokyo, Japan) with internal standards (arachidonic acid-d8 [Cayman Chemical], 15-hydroxyeicosatetraenoic acid-d4 [Cayman Chemical], and leukotriene B4-d5, [Cayman Chemical]). Briefly, the cartridge was washed with methanol and then water. The extracted sample in 50% methanol was then applied to the cartridge and washed with water and 50% methanol. The lipid sample was subsequently eluted by using 90% methanol containing 2% acetic acid.

### 2.11. LC–MS/MS Analysis of Free FAs and Their Metabolites

Lipid metabolites were analyzed by using a UPLC system (ACQUITY) (Waters, Milford, MA, USA) coupled with mass spectrometry (Orbitrap ELITE) (Thermo Fisher Scientific), with modifications of the previously reported protocol [29]. UPLC application was performed with a 1.7 mm, 1.0 × 150 mm ACQUITY UPLC BEH C18 column (Waters). Mass spectrometric analysis for quantification was based on the ion trap MS2 detection method. Data analysis was performed by using the software Xcalibur 2.2 (Thermo Fisher Scientific).

For quantification, calibration curves were drawn by using the following lipid standards: LA (Cayman Chemical), ALA (Cayman Chemical), ARA (Cayman Chemical), EPA (Cayman Chemical), DHA (Cayman Chemical), 18-hydroxyeicosapentaenoic acid (18-HEPE; Cayman Chemical), 15-HEPE (Cayman Chemical), 12-hydroxyeicosapentaenoic acid (12-HEPE; Cayman Chemical), 5-hydroxyeicosapentaenoic acid (5-HEPE; Cayman Chemical), and 17,18-epoxyeicosatetraenoic acid (17,18-EpETE; Cayman Chemical).

### 2.12. Reverse Transcription and Quantitative PCR

Reverse transcription and quantitative PCR analysis were performed as described previously [30]. In brief, RNA from cell suspensions was isolated by using Sepazol (Nacalai Tesque) and chloroform (Nacalai Tesque). After precipitation with 2-propanol (Nacalai Tesque) and washing with 75% (*vol*/*vol*) ethanol (Nacalai Tesque), the residue was incubated with DNaseI (Thermo Fisher Scientific) and reverse-transcribed to cDNA (Superscript 3 reverse transcriptase, VIRO cDNA Synthesis Kit; Invitrogen).

Real-time PCR was performed by using the LightCycler 480 System II (Roche, Basel, Switzerland) and SYBR Green I Master reagents (Roche). Primer sequences were: 5′-ggggatggagaagctacagg-3′ (sense) and 5′-tccgcttcaaacagagtgc-3′ (anti-sense) for *Alox15*, 5′-gaaagacaacggacaaatcacc-3′ (sense) and 5′-gggggtgatatgtttgaacttg-3′ (anti-sense) for *Pparg* and 5′-aaggccaaccgtgaaaagat-3′ (sense) and 5′-gtggtacgaccagaggcatac-3′ (anti-sense) for *Actb*.

### 2.13. Statistical Analysis

Statistical significance was evaluated by using one-way ANOVA followed by Dunn’s Kruskal–Wallis Multiple Comparisons or Tukey’s multiple comparison test for multiple groups and the Mann–Whitney test for 2 groups. In some analyses, outliers were excluded by the Smirnov–Grubbs test. All analyses were performed by using Prism 6 software (GraphPad Software, San Diego, CA, USA). A P value of less than 0.05 was considered significant.

## 3. Results

### 3.1. Dietary Linseed Oil Reduces Allergic Rhinitis Responses

**Figure 1:**
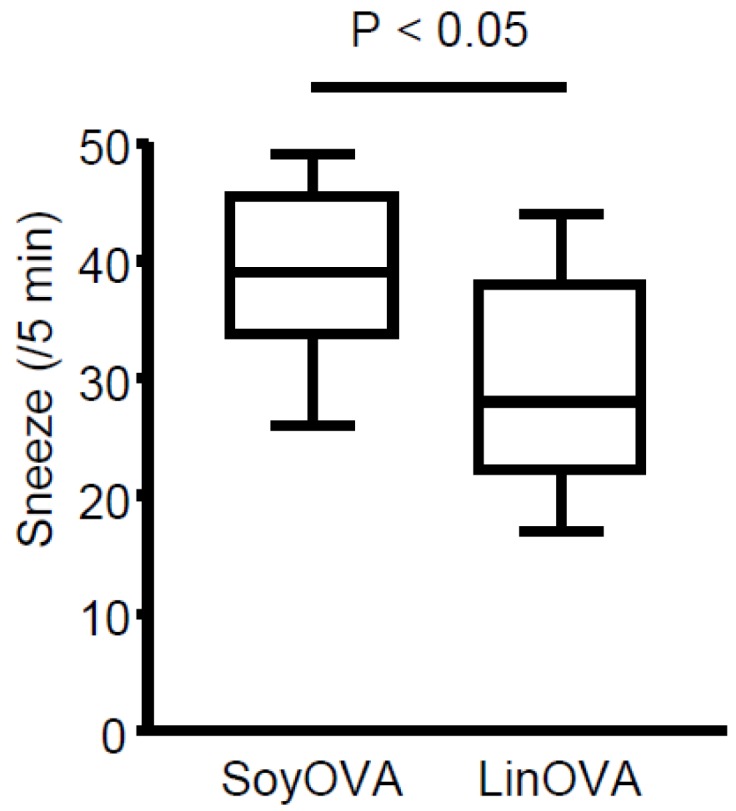
Dietary linseed oil reduces allergic rhinitis responses in mice. Mice were maintained on a diet containing 4% soybean oil (Soy) or linseed oil (Lin). After 2 months, ovalbumin (OVA)-induced allergic rhinitis was induced, and the sneezing rate was calculated. Data are from three independent experiments (*n* = 13). Center values indicate medians. Statistical significance was calculated by using the Mann–Whitney U test.

To clarify whether dietary linseed oil can ameliorate allergic rhinitis, mice were maintained on a diet containing linseed oil (omega-3 FA-enriched) or soybean oil (omega-6 FA-enriched; as a control dietary oil) (Table S1 and Figure S1). Subsequent administration of OVA induced allergic rhinitis and increased the frequency of sneezing in soybean oil-fed mice (SoyOVA). Compared with SoyOVA mice, the sneezing rate was decreased in linseed oil-fed mice (LinOVA) (Figure 1), suggesting that linseed oil reduced allergic responses.

### 3.2. Dietary FA Changes the FA Composition and Lipid Metabolites in NP and Plasma

**Figure 2:**
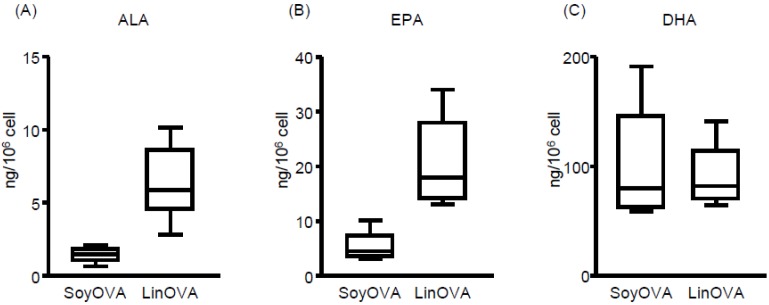
Dietary fatty acid is reflected in the fatty acid composition of the nasal passages of mice. (**A**–**C**) The OVA-induced allergic rhinitis model was applied to mice maintained on a diet containing 4% soybean oil (Soy) or linseed oil (Lin). Mice were challenged intranasally with OVA. Cells were collected from nasal passages, and the levels of (**A**) alpha-linolenic acid (ALA), (**B**) eicosapentaenoic acid (EPA), and (**C**) docosahexaenoic acid (DHA) in the cells were analyzed by using liquid chromatography coupled with tandem mass spectrometry (LC–MS/MS). Data are from two independent experiments (*n* = 7). Center values indicate medians.

To elucidate the underlying mechanism by which LinOVA mice showed a low frequency of sneezing, we analyzed the FA composition in the plasma and NP by using LC–MS/MS-based lipidomics. In plasma, omega-3 FA (ALA, EPA, and DHA) levels were increased in LinOVA mice, whereas omega-6 FA (LA and ARA) levels were increased in SoyOVA mice (Figure S2), consistent with our previous report [12]. In NP, we found that the LinOVA group contained higher amounts of omega-3 ALA and EPA, but not DHA, than the SoyOVA group (Figure 2). These results suggest that dietary FA can alter the FA composition in both the plasma and NP and that increased omega-3 FA levels in linseed oil-fed mice may provide an anti-allergic environment in the nose.

**Figure 3:**
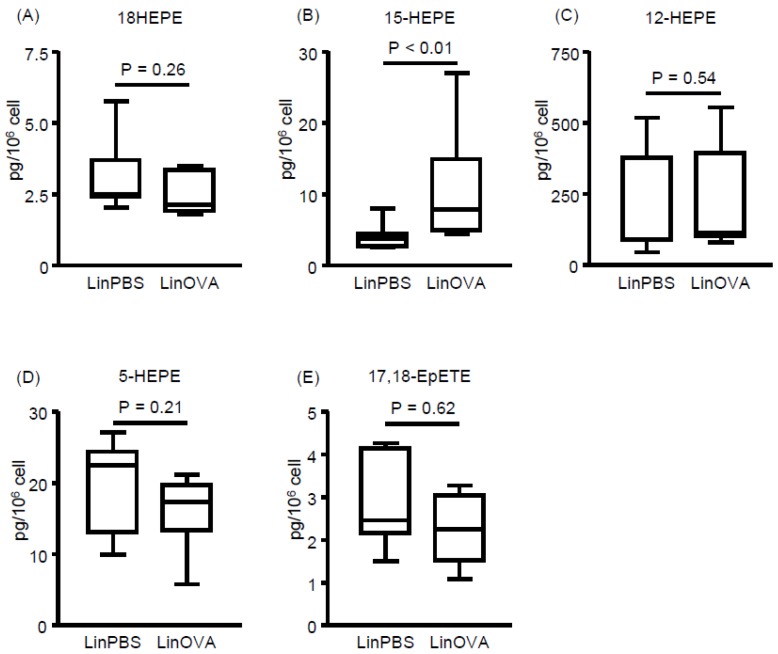
Induction of allergic rhinitis influences fatty acid metabolism in nasal passages. (**A**–**E**) EPA-derived fatty acid metabolites including (**A**) 18-hydroxyeicosapentaenoic acid (HEPE), (**B**) 15-HEPE, (**C**) 12-HEPE, (**D**) 5-HEPE, and (**E**) 17,18-epoxyeicosatetraenoic acid (17,18-EpETE) in nasal passages were analyzed by using LC–MS/MS. Data are from two independent experiments (*n* = 7). Center values indicate medians. Statistical significance between two groups was calculated by using the Mann–Whitney U test.

In our previous study, dietary linseed oil increased EPA-derived metabolite levels in the large intestine, where they alleviated food allergy-induced diarrhea [12]. This observation indicated that dietary linseed oil could change the composition of not only FAs but also their metabolites in the tissues, some of which then contributed to the prevention of allergic symptoms. Consistent with this concept, previous reports have suggested that some omega-3 FA-derived metabolites alleviate inflammation by resolving, but not preventing, the inflammation [31,32]. To test this hypothesis, we next analyzed EPA-derived metabolites in the NP and compared the concentrations between the non-allergic (LinPBS) and allergic (LinOVA) groups, as well as the SoyOVA group. Although the levels of various EPA-derived metabolites were increased in the LinOVA group relative to the SoyOVA group (Figure S3), 15-HEPE was selectively higher in the LinOVA group than in the LinPBS group, whereas no significant difference was found for the other EPA-derived metabolites (18-HEPE, 12-HEPE, 5-HEPE, and 17,18-EpETE) (Figure 3). Given that 15-HEPE levels in plasma did not differ significantly between the LinPBS and LinOVA groups (Figure S4), the difference between the dietary oils reflected the FA composition, with 15-HEPE levels being preferentially and specifically increased in the NP of the LinOVA group.

### 3.3. Nasal Administration of 15-HEPE Inhibits Allergic Rhinitis

**Figure 4:**
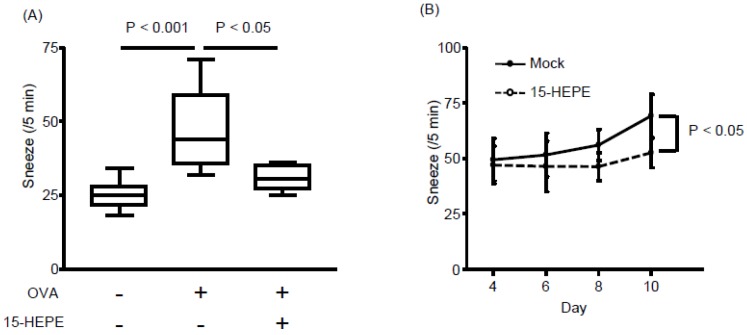
Nasal administration of 15-HEPE dampens allergic rhinitis. (**A**) Mice were nasally treated with 15-HEPE or vehicle (0.5% EtOH) before challenge in an allergic rhinitis model. Sneezing rates were calculated. Data are from two independent experiments (*n* = 8–10). Center values indicate medians. Statistical significance was calculated by using one-way ANOVA followed by Dunn’s Kruskal–Wallis Multiple Comparisons. (**B**) Allergic rhinitis was induced in mice during days 1 through 4. From day 5 through 10, 15-HEPE or mock treatment (0.5% EtOH PBS) was nasally administered 5 min before OVA challenge. Then the sneezing rate was calculated on days 4 through 10. Data are from three independent experiments (*n* = 11 or 12). Data are expressed as the mean ± SEM. Statistical significance was calculated by using the Mann–Whitney U test.

The unique increase in 15-HEPE levels in the LinOVA group prompted us to determine whether 15-HEPE has anti-rhinitis effects. When synthetic 15-HEPE was nasally administered from the first day of OVA challenge, sneezing frequency decreased compared with that in mock-treated controls (Figure 4A). Because the 15-HEPE level increased after the induction of allergic rhinitis (Figure 3), 15-HEPE could exert a therapeutic effect. Accordingly, we similarly treated mice in which allergic rhinitis had already been induced. After inducing allergic rhinitis by administering OVA for consecutive 4 days, treatment with 15-HEPE or vehicle was initiated on day 5 until day 10 at which time the mice were challenged OVA again. The 15-HEPE-treated group showed little increase in sneezing frequency, whereas the sneezing frequency increased significantly in the mock-treated group (Figure 4B). These results show that 15-HEPE exerts protective effects against allergic rhinitis.

### 3.4. Eosinophils Express High Levels of 15-lipoxygenase (15-LOX) and Preferentially Produce 15-HEPE from EPA

**Figure 5:**
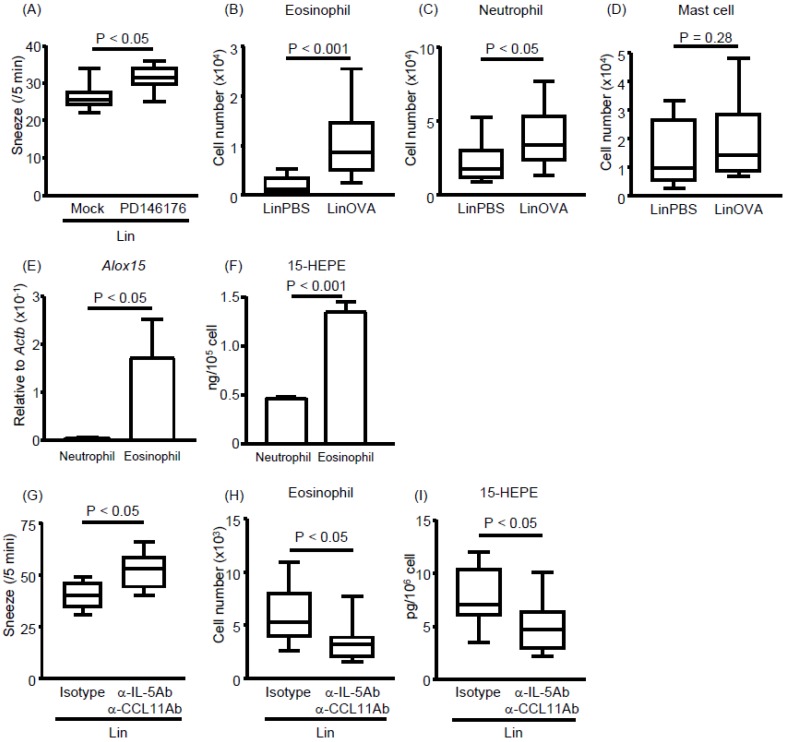
Eosinophils infiltrate the nasal passages upon the development of allergic rhinitis and produce 15-HEPE and eosinophil inhibition aggravates allergic rhinitis in linseed oil-fed mice. (**A**) PD146176, an inhibitor of 15-LOX, was injected into mice fed a linseed oil diet, and sneezing rates were calculated after the induction of allergic rhinitis. Data are from two independent experiments (*n* = 8). Center values indicate medians. Statistical significance was calculated by using the Mann–Whitney U test. (**B**–**D**) After the induction of allergic rhinitis, the numbers of (**B**) eosinophils, (**C**) neutrophils, and (**D**) mast cells in the nasal passage were calculated based on the total cell number and Flow cytometry data. Data are from three independent experiments (*n* = 9 or 10). Center values indicate medians. Statistical significance was calculated by using the Mann–Whitney U test. (**E**) Quantitative PCR analysis was performed to measure *Alox15* mRNA expression in neutrophils and eosinophils isolated from nasal passages. Data are expressed as the mean ± SEM (*n* = 8 or 9). Statistical significance was calculated by using the Mann–Whitney U test. (**F**) Bone marrow-derived neutrophils and eosinophils were stimulated with 2 μM calcium ionophore in the presence of 1 μM EPA or ARA. After 30 min, the 15-HEPE level in the supernatant was analyzed by using LC–MS/MS. The panel shows representative data from two experiments with similar results (*n* = 4). Data are expressed as the mean ± SEM. Statistical significance was calculated by using the Mann–Whitney U test. (**G**–**I**) A mixture of anti-IL-5 antibody and anti-CCL11 antibody was injected into mice fed a linseed oil diet. Rat-IgG1 isotype was used as an isotype control. Allergic rhinitis was induced and (**G**) the sneezing rate, (**H**) eosinophil number in the NP, and (**I**) 15-HEPE level in the NP were analyzed. Data are from two independent experiments (*n* = 8). Center values indicate medians. Statistical significance was calculated by using the Mann–Whitney U test.

Lipidomic analysis revealed that 15-HEPE levels increased with the induction of allergic rhinitis in the NP of linseed oil-fed mice (Figure 3). Because 15-HEPE is generated by 15-LOX, we evaluated whether 15-LOX inhibition affects allergic symptoms in linseed oil-fed mice. Administration of a 15-LOX inhibitor increased the frequency of sneezing (Figure 5A), indicating an association between 15-LOX-mediated omega-3 fatty acid metabolism and the regulation of rhinitis.

We next focused on identifying which cell type expressed high levels of 15-LOX. We predicted that the 15-HEPE-producing cell migrated into the NP upon allergy induction because the 15-HEPE level was increased upon allergic rhinitis induction only in the NP (Figure 3) and not in the plasma (Figure S3). Therefore, we compared the numbers of cells that migrated into the NP after allergy induction. We found that neutrophil and eosinophil numbers were increased in allergic mice compared with control mice, whereas mast cell numbers were unchanged (Figure 5B–D). We then isolated neutrophils and eosinophils from the NP and measured their gene expression levels of 15-LOX. The 15-LOX expression was higher in eosinophils than neutrophils (Figure 5E), suggesting that eosinophils were the main producers of 15-HEPE in linseed oil-fed mice experiencing allergic rhinitis. To test this possibility, we assessed the lipid metabolism of eosinophils and neutrophils in vitro. When bone marrow-derived eosinophils or neutrophils were activated with a calcium ionophore in the presence of EPA, the eosinophils produced more 15-HEPE than did the neutrophils (Figure 5F). These results suggest that eosinophils infiltrate the NP, where they regulate allergic rhinitis by producing 15-HEPE from EPA.

We next examined whether depletion of eosinophils in vivo aggravates nasal rhinitis. To test this, we intravenously treated LinOVA mice with a mixture of anti-IL-5 antibody and anti-CCL11 antibody and induced allergic rhinitis in the model. Antibody administration increased the frequency of sneezing accompanied by a reduction of both eosinophil numbers and 15-HEPE levels in the NP (Figure 5G–I). These results suggest that eosinophils exert anti-rhinitis effects through the production of 15-HEPE from EPA in the NP.

### 3.5. The 15-HEPE Interacts with PPARγ and Inhibits Mast Cell Degranulation

**Figure 6:**
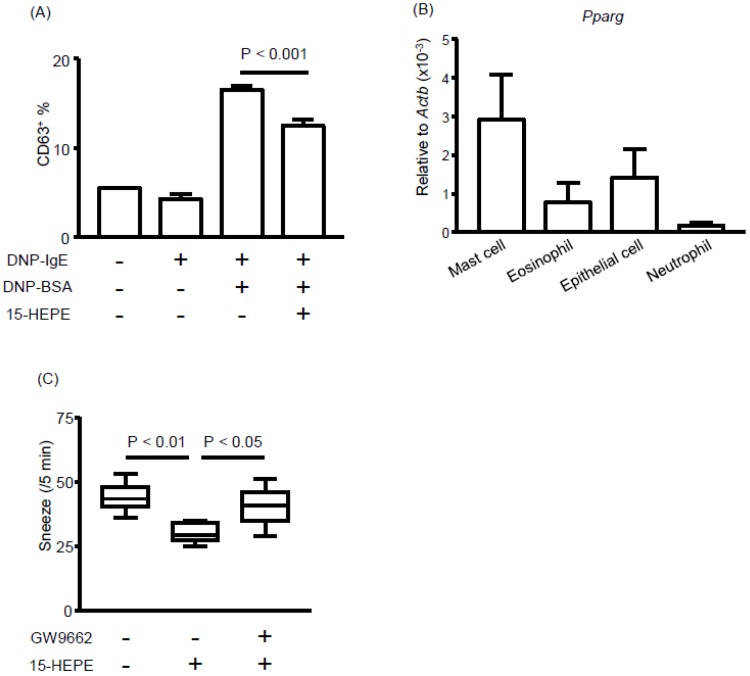
The 15-HEPE interacts with peroxisome proliferator-activated receptor gamma (PPARγ) and inhibits mast cell degranulation. (**A**) In vitro degranulation of mast cells was induced in the presence or absence of 15-HEPE in vitro. Degranulation was determined by CD63 expression by using FACS. Data are from two independent experiments (*n* = 4 for DNP-BSA-untreated groups and *n* = 8 for dinitrophenyl [DNP]-BSA-treated groups) and expressed as the mean ± SEM. Statistical significance was calculated by using one-way ANOVA followed by Tukey’s Multiple Comparisons. (**B**) Mast cells, eosinophils, epithelial cells, and neutrophils were isolated from mice NP and *Pparg* gene expression was assessed. Data are from two independent experiments (*n* = 8) and are expressed as the mean ± SEM. (**C**) GW9662, an antagonist of PPARγ, was injected into mice with allergic rhinitis 30 min before 15-HEPE administration. Sneezing rates were calculated. Data are from two independent experiments (*n* = 8). Center values indicate medians. Statistical significance was calculated by using one-way ANOVA followed by Dunn’s Kruskal–Wallis Multiple Comparisons.

Next, we asked how 15-HEPE exerts its anti-allergic effect. Because the OVA-specific plasma IgE concentration did not differ significantly between the 15-HEPE- and mock-treated groups (Figure S5), we focused on mast cell degranulation, another target of allergy inhibition. We first evaluated whether 15-HEPE inhibited mast cell degranulation by using bone marrow-derived mast cells and found that 15-HEPE reduced mast cell degranulation in vitro (Figure 6A). We next focused on the target receptor of 15-HEPE on mast cells. Of the several FA receptors, we focused on PPARγ because administration of a PPARγ agonist has been shown to inhibit allergic rhinitis by suppressing mast cell functions [33,34]. We checked the gene expression of PPARγ on each cell type isolated from the NP of allergic mice. Among the four types of cell examined, mast cells showed high levels of PPARγ expression compared with those of eosinophils, epithelial cells, and neutrophils (Figure 6B). To test whether the anti-rhinitis effect of 15-HEPE was exerted through an interaction with PPARγ in vivo, a PPARγ antagonist (GW9662) was administered to the mice before 15-HEPE administration, resulting in the nullification of the anti-allergic activity of 15-HEPE (Figure 6C). These results collectively suggest that 15-HEPE dampens allergic rhinitis by inhibiting mast cell degranulation through an interaction with PPARγ.

## 4. Discussion

Numerous studies suggest that dietary FA affects health and diseases. In particular, omega-3 FA and its metabolites have several beneficial functions including anti-hypertension [35], anti-metabolic syndrome [36] and anti-allergy functions [12,13,37]. In the current study, we investigated the effect of dietary omega-3 FA on allergic rhinitis in mice. We found that dietary linseed oil dampened allergic rhinitis response in mice. Lipidomics revealed that ALA and EPA were increased in linseed oil-fed mice and 15-HEPE was accumulated in NP of linseed oil-fed mice after the induction of nasal allergy. The 15-HEPE was produced by the 15-LOX activity of eosinophil which infiltrated into NP and deletion of eosinophil exacerbated allergic responses in linseed oil-fed mice. The 15-HEPE reduces mast cell degranulation in vitro and dampened allergic responses in mice in a PPARγ-dependent manner.

Type I allergy is initiated by the production of allergen-specific IgE and subsequently mediated via cross-linking with FcεRI on mast cells accompanied by degranulation and release of chemical mediators [3]. In this study, we found that 15-HEPE levels were increased by dietary intake of linseed oil and exerted an anti-allergic effect by inhibiting mast cell degranulation without affecting allergen-specific IgE production. The inhibition of mast cell degranulation is among the most common strategies to ameliorate type I allergic symptoms, such as those associated with atopic dermatitis [34], food allergy [12], and rhinitis [38]. Previous reports indicated that a PPARγ agonist inhibited atopic dermatitis in mice through the suppression of mast cell maturation and mediator release [34,39]. In the present study, we found that mast cells in the NP of allergic mice expressed PPARγ, which acts as a receptor for 15-HEPE to inhibit degranulation in vitro.

We also found that the induction of allergic rhinitis changed FA metabolism in the NP. A previous study reported that some anti-inflammatory lipid mediators were endogenously generated during the resolution phase of zymosan-initiated peritonitis and acted as resolving molecules [31]. In the present study, EPA-derived 15-HEPE levels increased in linseed oil-fed mice with allergic rhinitis.

Increased 15-HEPE levels were found in the NP but not in the plasma of LinOVA. Several reports have indicated that FA metabolism differs between tissues. For example, EPA-derived 17,18-EpETE uniquely accumulates in the large intestine in linseed oil-fed mice [12]. Another report suggested that 12-hydroxyeicosatetraenoic acid and 13-hydroxyoctadecadienoic acid levels were preferentially increased in the lungs of asthmatic mice [40]. These differences can be explained, at least in part, by the cells that express the responsible enzymes. Indeed, macrophages in the heart of *fat-1* transgenic mice produce 18-HEPE, which inhibits macrophage-mediated proinflammatory activation of cardiac fibroblasts [41]. The 18-HEPE is further transformed to resolvin E1 by 5-LOX expressed on neutrophils [42,43]. We also recently showed that neutrophil 5-LOX contributes to the production of leukotriene B_4_ from ARA and influences the development of inflammation [26].

In the present study, we found that eosinophils highly express 15-LOX. Generally, eosinophil numbers are considered to reflect the severity of the allergic symptoms of asthma and rhinitis. Indeed, eosinophils release cytotoxic proteins, including major basic protein (MBP), eosinophil cationic protein (ECP), and eosinophil peroxidase (EPx), which exacerbate inflammation [44]. In addition, eosinophils are recognized as activators of mast cells and basophils [45,46]. In contrast, some recent studies have investigated the anti-inflammatory activities of eosinophils. These studies have revealed that eosinophils also regulate infectious bacteria-induced intestinal inflammation by suppressing Th1 responses [47]. In the lung, pulmonary resident eosinophils have regulatory profiles against Th2 responses [48]. Moreover, recent studies have revealed that eosinophils participate in the lipid network for the production of anti-inflammatory and/or pro-resolution lipid mediators [49,50,51]. It is also indicated that eosinophils may be involved in the control of nasal allergy, but details on the mechanisms have been unclear [52]. In the present study, we demonstrated the novel function of eosinophils in the control of allergic responses. We found that eosinophils infiltrated the NP upon the induction of allergic rhinitis and produced 15-HEPE for the inhibition of mast cell degranulation. Thus, eosinophils produce several types of lipid mediators for the regulation of inflammation and allergy by targeting different types of immune cells such as neutrophils, phagocytes and mast cells. Eosinophil infiltration occurs regardless of dietary oils; however, the intake of linseed oil resulted in an EPA-enriched environment, where eosinophils metabolized EPA to produce 15-HEPE for the inhibition of allergic rhinitis responses.

In conclusion, our findings suggest that ALA, which is abundant in linseed oil, reduces allergic responses through the local production of 15-HEPE by eosinophils that infiltrate the NP. The 15-HEPE dampens allergic responses in a PPARγ-dependent manner and inhibits mast cell degranulation. These findings identify unique eosinophil functions in the development of allergic rhinitis which are determined by the surrounding lipid environment. Our study also provides valuable information for both dietary and pharmaceutical strategies to ameliorate allergic symptoms.

## Supplementary Materials

The following are available online at https://www.mdpi.com/2072-6643/11/12/2868/s1, Figure S1: Fatty acid composition of Soybean oil and Linseed oil, Figure S2: Effect of dietary oils on the plasma FA composition, Figure S3: Dietary fatty acids influence the accumulation of fatty acid metabolites in the NP, Figure S4: 15-HEPE concentration in plasma, Figure S5: OVA-specific IgE concentration in plasma, Table S1: Composition of diet.

## Abbreviations

ALA	alpha-linolenic acid
ARA	Arachidonic acid
CD	Cluster of differentiation
COX	Cyclooxygenase
CYP	Cytochrome P450
DHA	Docosahexaenoic acid
DMSO	Dimethyl sulfoxide
EPA	Eicosapentaenoic acid
EpETE	Epoxyeicosatetraenoic acid
FA	Fatty acid
LA	Linoleic acid
LC–MS/MS	Liquid chromatography coupled with tandem mass spectrometry
LOX	Lipoxygenase
NP	Nasal passage
OVA	Ovalbumin
PPAR	Peroxisome proliferator-activated receptor gamma
