# Supplementary material for: Dietary Omega-3 Fatty Acid Dampens Allergic Rhinitis via Eosinophilic Production of the Anti-Allergic Lipid Mediator 15-Hydroxyeicosapentaenoic Acid in Mice
Source: Nutrients. 2019 Nov 22;11(12):2868. doi: 10.3390/nu11122868 (PMC6950470; doi:10.3390/nu11122868)
Supplement: Supplementary file 1 [file nutrients-11-02868-s001.zip › nutrients-633037-supplementary.pptx]

## Slide 1
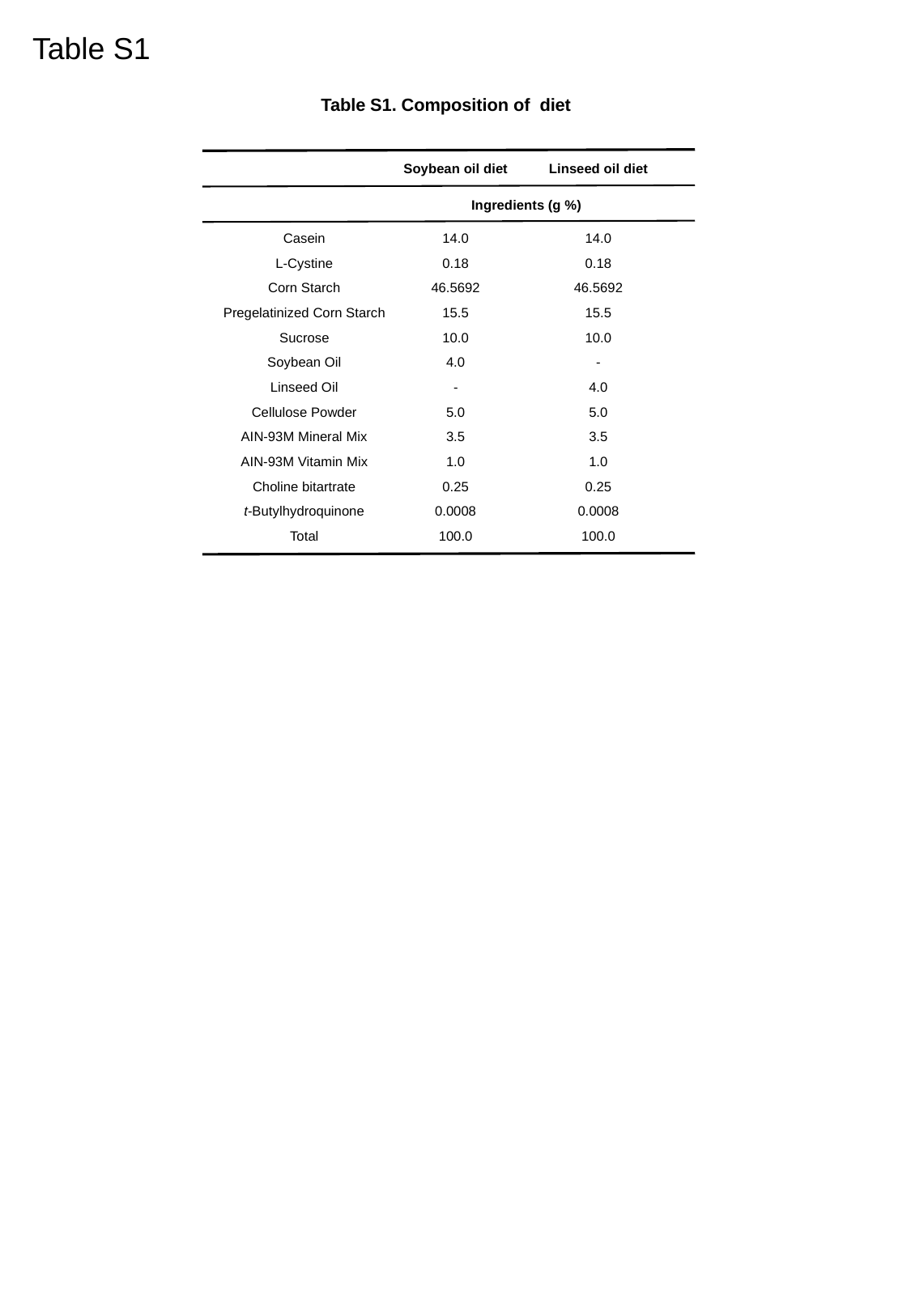

Table S1
Table S1. Composition of diet
Soybean oil diet
Linseed oil diet
Ingredients (g %)
Casein
14.0
14.0
L-Cystine
0.18
0.18
Corn Starch
46.5692
46.5692
Pregelatinized Corn Starch
15.5
15.5
Sucrose
10.0
10.0
Soybean Oil
4.0
-
-
Linseed Oil
4.0
Cellulose Powder
5.0
5.0
AIN-93M Mineral Mix
3.5
3.5
AIN-93M Vitamin Mix
1.0
1.0
Choline bitartrate
0.25
0.25
t-Butylhydroquinone
0.0008
0.0008
Total
100.0
100.0

## Slide 2
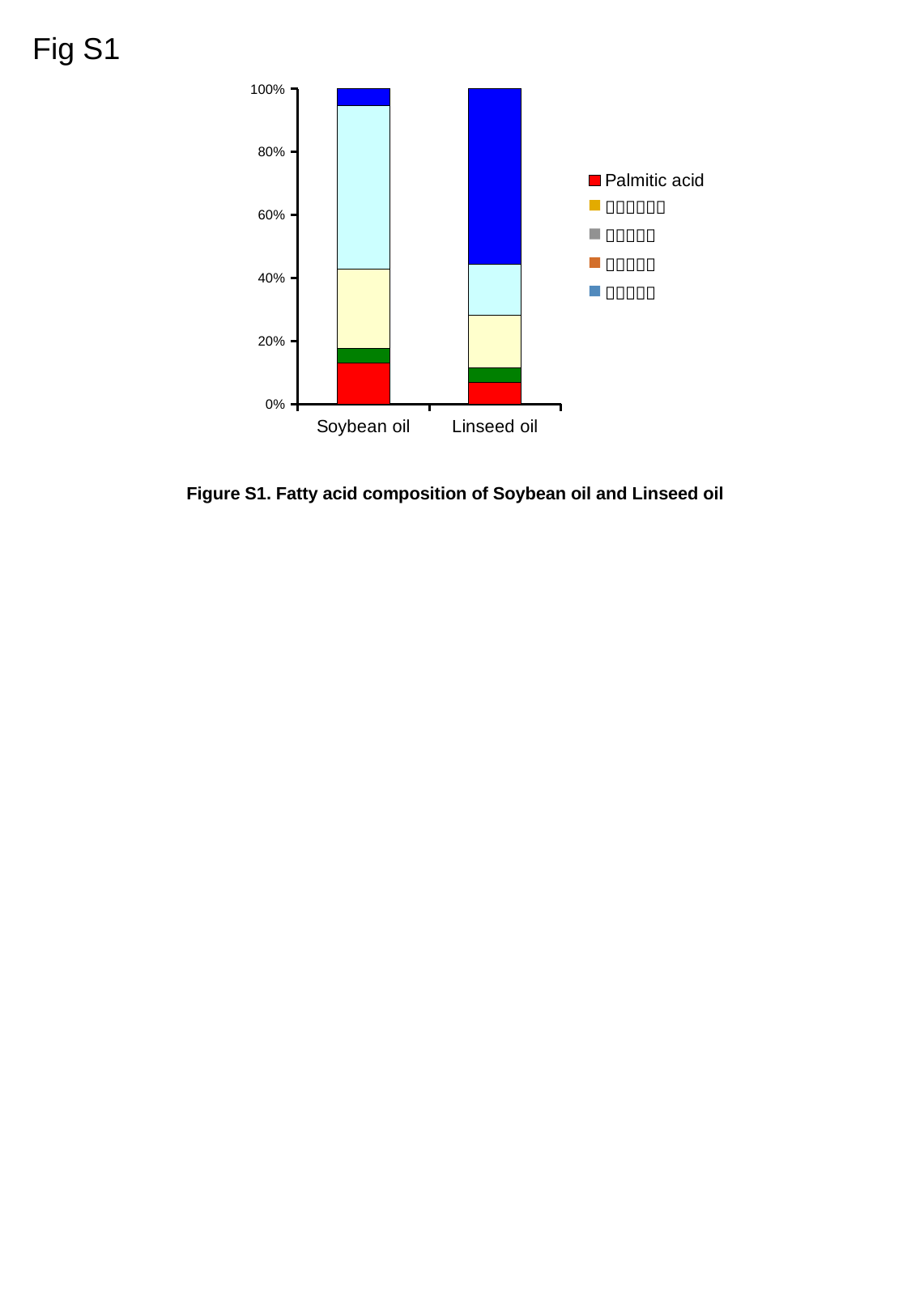

Fig S1
### Chart
| Category | カプリル酸 | カプリン酸 | ラウリン酸 | ミリスチン酸 | Palmitic acid | Stearic acid | Oleic acid | Linoleic acid | α-linolenic acid |
|---|---|---|---|---|---|---|---|---|---|
| Soybean oil | 0.0 | 0.0 | 0.0 | 0.0 | 13.029315960912053 | 4.5602605863192185 | 25.0814332247557 | 52.11726384364821 | 5.211726384364821 |
| Linseed oil | 0.0 | 0.0 | 0.0 | 0.0 | 6.796116504854369 | 4.53074433656958 | 16.8284789644013 | 16.181229773462785 | 55.663430420711975 |Figure S1. Fatty acid composition of Soybean oil and Linseed oil

## Slide 3
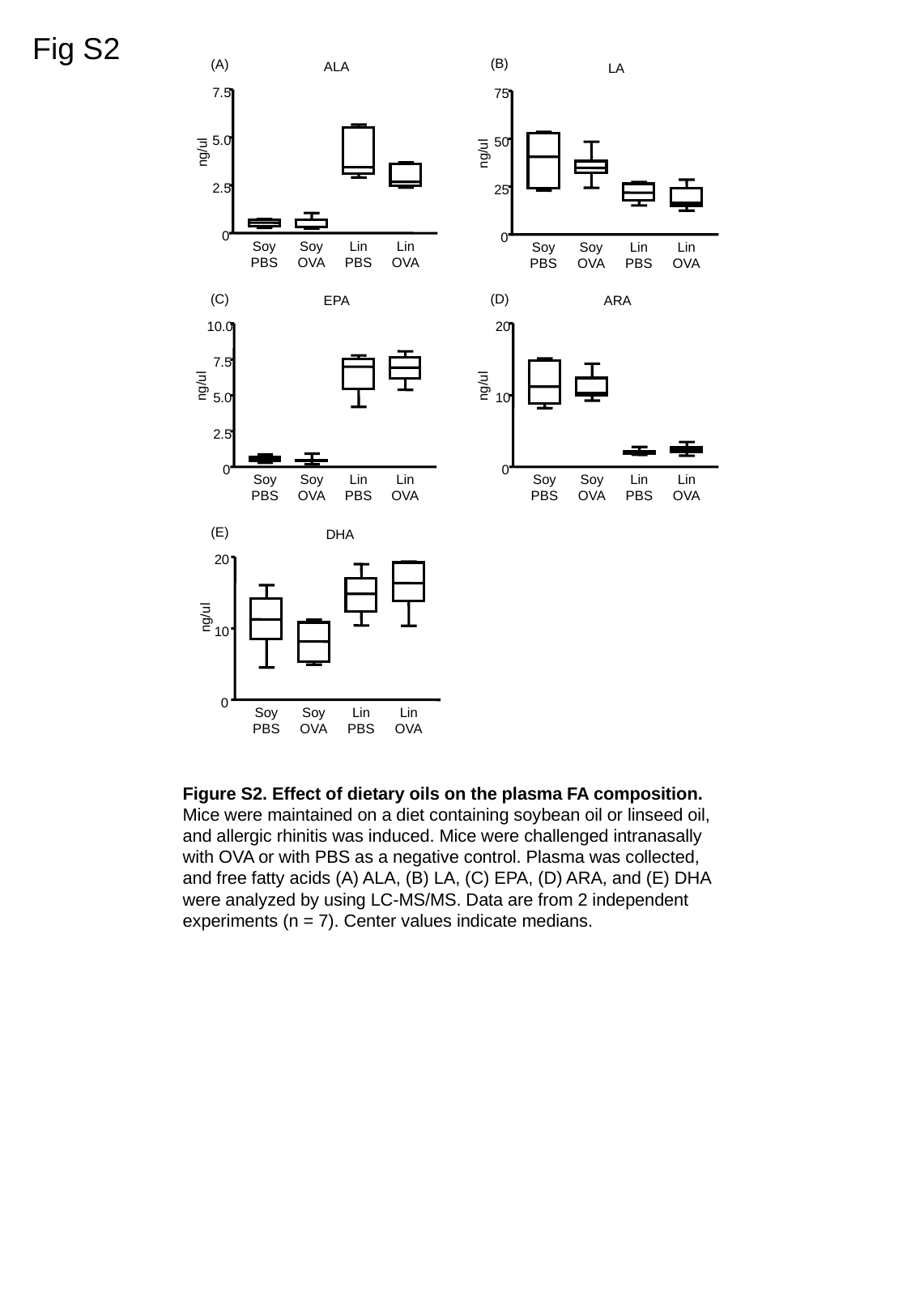

Fig S2
(B)
(A)
ALA
7.5
5.0
ng/ul
2.5
0
Soy
PBS
Soy
OVA
Lin
PBS
Lin
OVA
LA
75
50
ng/ul
25
0
Soy
PBS
Soy
OVA
Lin
PBS
Lin
OVA
EPA
10.0
7.5
ng/ul
5.0
2.5
0
Soy
PBS
Soy
OVA
Lin
PBS
Lin
OVA
ARA
20
ng/ul
10
0
Soy
PBS
Soy
OVA
Lin
PBS
Lin
OVA
DHA
20
ng/ul
10
0
Soy
PBS
Soy
OVA
Lin
PBS
Lin
OVA
(C)
(D)
(E)
Figure S2. Effect of dietary oils on the plasma FA composition.
Mice were maintained on a diet containing soybean oil or linseed oil, and allergic rhinitis was induced. Mice were challenged intranasally with OVA or with PBS as a negative control. Plasma was collected, and free fatty acids (A) ALA, (B) LA, (C) EPA, (D) ARA, and (E) DHA were analyzed by using LC-MS/MS. Data are from 2 independent experiments (n = 7). Center values indicate medians.

## Slide 4
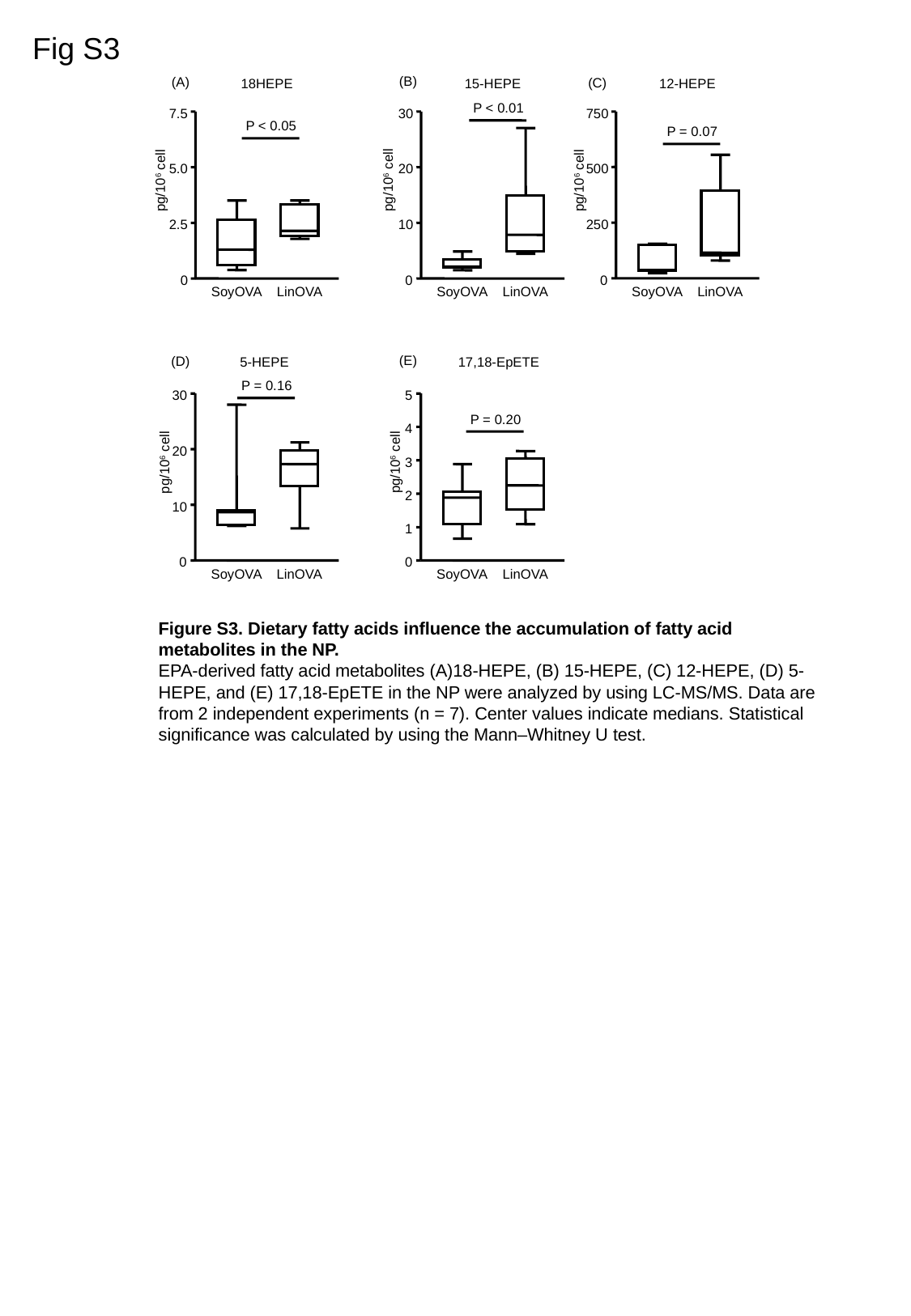

Fig S3
(B)
(A)
(C)
18HEPE
7.5
P < 0.05
5.0
pg/106 cell
SoyOVA
LinOVA
2.5
0
12-HEPE
750
P = 0.07
LinOVA
500
pg/106 cell
250
SoyOVA
0
15-HEPE
P < 0.01
30
LinOVA
20
pg/106 cell
10
SoyOVA
0
5-HEPE
P = 0.16
30
SoyOVA
20
LinOVA
pg/106 cell
10
0
17,18-EpETE
5
P = 0.20
4
LinOVA
3
pg/106 cell
SoyOVA
2
1
0
(E)
(D)
Figure S3. Dietary fatty acids influence the accumulation of fatty acid metabolites in the NP.
EPA-derived fatty acid metabolites (A)18-HEPE, (B) 15-HEPE, (C) 12-HEPE, (D) 5-HEPE, and (E) 17,18-EpETE in the NP were analyzed by using LC-MS/MS. Data are from 2 independent experiments (n = 7). Center values indicate medians. Statistical significance was calculated by using the Mann–Whitney U test.

## Slide 5
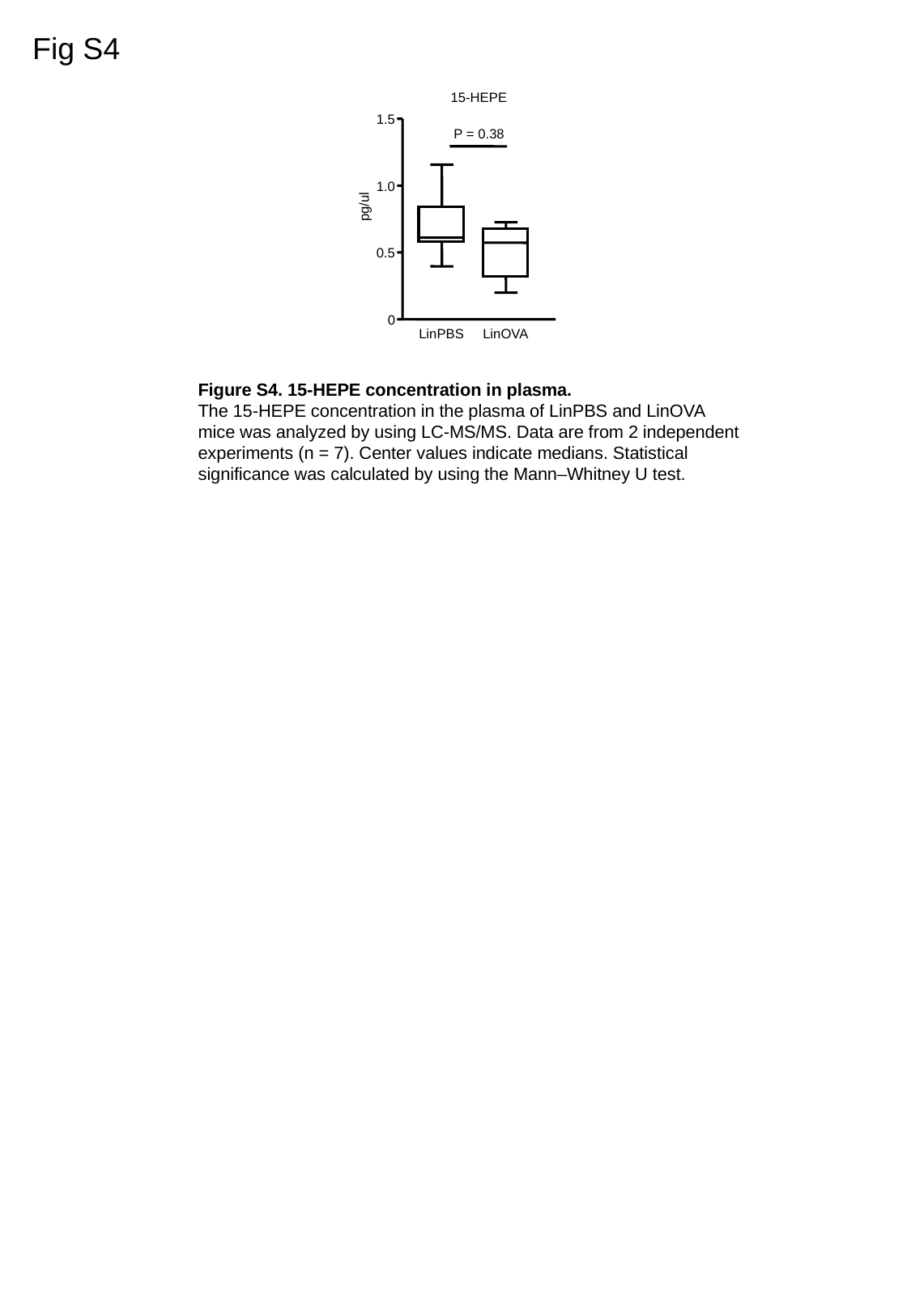

Fig S4
15-HEPE
1.5
P = 0.38
LinPBS
1.0
pg/ul
LinOVA
0.5
0
Figure S4. 15-HEPE concentration in plasma.
The 15-HEPE concentration in the plasma of LinPBS and LinOVA mice was analyzed by using LC-MS/MS. Data are from 2 independent experiments (n = 7). Center values indicate medians. Statistical significance was calculated by using the Mann–Whitney U test.

## Slide 6
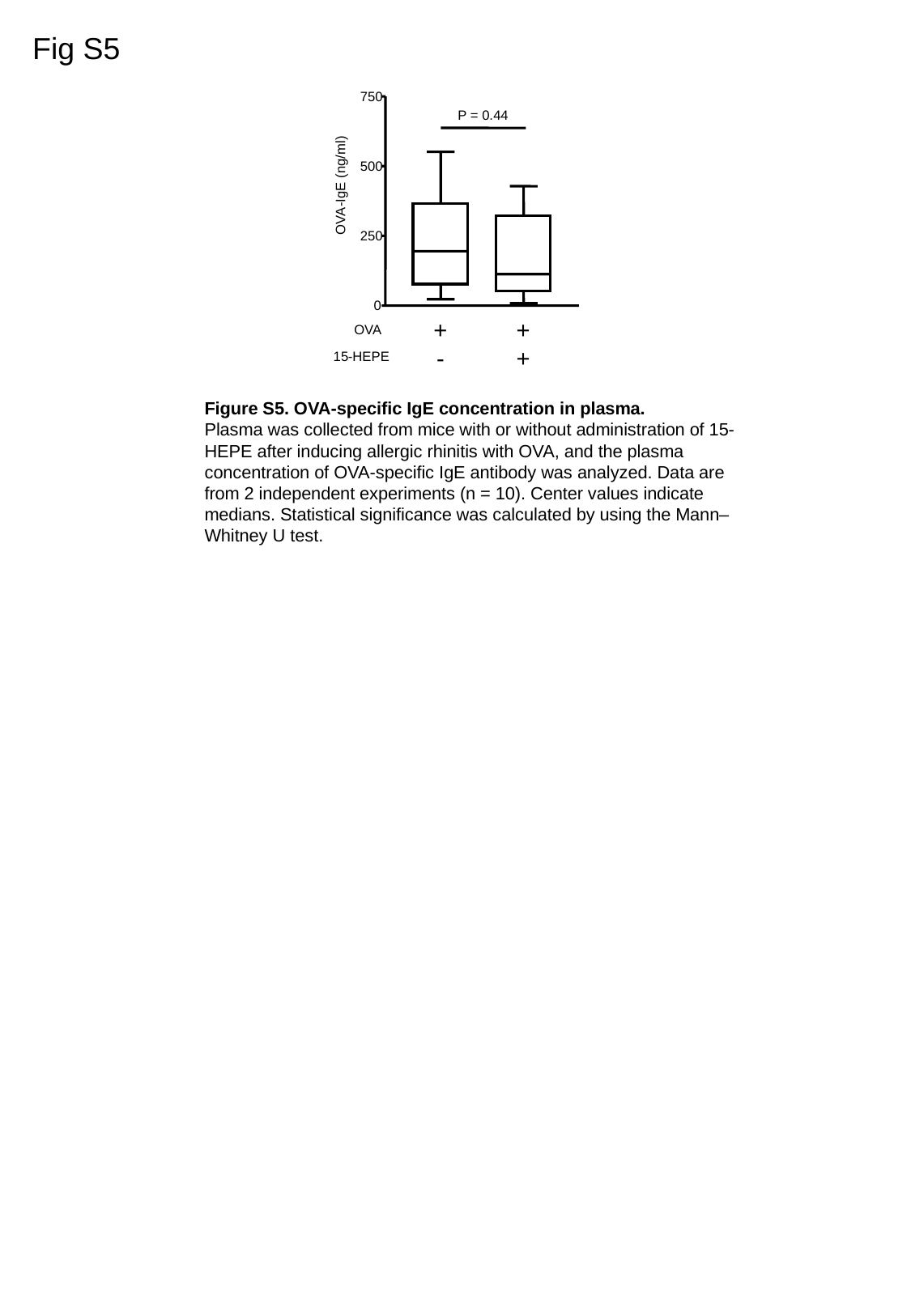

Fig S5
750
P = 0.44
500
OVA-IgE (ng/ml)
250
0
+
+
OVA
-
+
15-HEPE
Figure S5. OVA-specific IgE concentration in plasma.
Plasma was collected from mice with or without administration of 15-HEPE after inducing allergic rhinitis with OVA, and the plasma concentration of OVA-specific IgE antibody was analyzed. Data are from 2 independent experiments (n = 10). Center values indicate medians. Statistical significance was calculated by using the Mann–Whitney U test.
